# Neurologic Disorders and Hepatitis E, France, 2010

**DOI:** 10.3201/eid1708.102028

**Published:** 2011-08

**Authors:** Laura-Anne Despierres, Elsa Kaphan, Shahram Attarian, Stephan Cohen-Bacrie, Jean Pelletier, Jean Pouget, Anne Motte, Rémi Charrel, René Gerolami, Philippe Colson

**Affiliations:** Author affiliations: Centre Hospitalo-Universitaire Timone, Marseille, France (L.-A. Despierres, E. Kaphan, S. Attarian, S. Cohen-Bacrie, J. Pelletier, J. Pouget , A. Motte, R. Charrel, P. Colson);; Université de la Méditerranée, Marseille (S. Cohen-Bacrie, R. Charrel, P. C*o*lson);; Centre Hospitalo-Universitaire Conception, Marseille (R. Gerolami)

**Keywords:** Acute hepatitis E, hepatitis E virus, neurologic symptoms, meningitis, polyradiculoneuropathy, France, autochthonous hepatitis, viruses, dispatch

## Abstract

We report meningitis with diffuse neuralgic pain or polyradiculoneuropathy associated with PCR-documented acute hepatitis E in 2 adults. These observations suggest that diagnostic testing for hepatitis E virus should be conducted for patients who have neurologic symptoms and liver cytolysis.

Hepatitis E virus (HEV), a major cause of acute hepatitis in tropical and subtropical countries, is emerging in industrialized countries, where an increasing number of autochthonous hepatitis E cases have been reported (*1**–**5*). Nonetheless, the clinical spectrum of HEV infection still might be incompletely characterized. During the past decade, 12 cases of hepatitis E with neurologic signs and symptoms have been reported (*1**–**4**,**6**–**13*). We report 2 cases of meningitis or polyradiculoneuropathy in association with PCR-documented acute hepatitis E in France during 2010.

## The Cases

Case 1 occurred in a 54-year-old woman hospitalized in August 2010 for subacute diffuse paresthesia and pain of the limbs (with ulnar localization on upper limbs and proximal pain in legs), headaches, cervicalgia, photophonophobia, transient fever, and nausea. Reflexes were all present, without pyramidal signs. No abnormalities were found by medullar magnetic resonance imaging. Nerve conduction study was not done because of rapid clinical improvement beginning <1 week after onset. The patient had no notable medical history. At admission, alanine aminotransferase level was 566 IU/L (reference values 8–34 IU/L), γ-glutamyl transferase level was 184 IU/L (reference 9–38 IU/L), alkaline phosphatase level was 125 IU/L (reference 42–98 IU/L), and bilirubin was 11 µmol/L (reference 5–34 µmol/L). Cerebrospinal fluid (CSF) was clear; protein and glucose levels were 1.03 g/L (reference 0.15–0.45) and 3.4 mmol/L (reference 3.0–4.5 mmol/L), respectively; leukocyte count was 74 cells/mm^3^, including 90% mononuclear cells; erythrocyte count was 1 cell/mm^3^. Immunoelectrophoresis performed in CSF and serum indicated rupture of the blood–brain barrier. Antibiotherapy with ceftriaxone, amoxicillin, and acyclovir was introduced 2 days after admission and continued for 2 weeks. CSF culture remained sterile at day 5.

Several infectious causes for neurologic symptoms and acute hepatitis were excluded by nucleic acid and serologic assays performed on serum and CSF (Table A1). In contrast, HEV infection was diagnosed by detection of anti-HEV immunoglobulin (Ig) M (cutoff optical density ratio >10; threshold value 1 [Adaltis, Rome, Italy]) and by detection of HEV RNA in serum (*5*). Concurrently, anti-HEV IgM (Assure, MP Diagnostics, Illkirch, France) and HEV RNA were detected in CSF. HEV RNA levels measured by real-time PCR were ≈2 log greater in the serum (cycle threshold [C_t_] 27) than in CSF (C_t_ 33). HEV RNA open reading frame 2 sequences were recovered from serum (GenBank accession nos. HQ702487 and HQ702490) and CSF (GenBank accession no. HQ702488) by using in-house protocols (*14*); they showed 100% nt identity with each other. Phylogenetic analysis identified genotype 3, which is commonly found in autochthonous cases in France (*4**,**5*). No source for HEV transmission was identified, including eating undercooked pork or drinking unsafe water; the patient had returned from Greece 3 weeks before her hospitalization. She recovered fully within 2 weeks after onset of hepatitis and neurologic disorders. Liver parameters returned to within normal limits within 2 weeks, and HEV RNA was cleared in serum after 3 weeks.

Case 2 occurred in a 49-year-old man hospitalized in July 2010 for acute proximal sensorimotor weakness of the limbs. Clinical examination showed proximal muscle weakness associated with paresthesia and pain of upper and lower limbs. Muscle stretch reflexes were diminished in the lower limbs. No pyramidal signs were evident. Nerve conduction study results were normal, and needle electromyography showed abnormal spontaneous activity and neurogenic recruitment in weak muscles. The diagnosis of acute inflammatory polyradiculoneuropathy was retained. Liver enzymes were elevated: alanine aminotransferase 78 IU/L (reference <60 IU/L) and γ-glutamyl transferase 81 IU/L (reference <60 IU/L); bilirubin levels were within reference range. CSF was macroscopically clear; protein and glucose levels were 1.02 g/L (reference 0.15–0.45 g/L) and 3.6 mmol/L (reference 2.2–3.9 mmol/L), respectively. Leukocyte count was 10 cells/mm^3^, including 87% lymphocytes; erythrocyte count was 3 cells/mm^3^. CSF culture was sterile after 5 days. As in case 1, several infectious etiologies were excluded (Table A1). Anti-HEV IgM was detected in serum (optical density ratio >10) but not in CSF. HEV RNA was detected by real-time PCR only in serum (C_t_ 34). HEV RNA was of genotype 3 (GenBank accession no. HQ702489); nucleotide identity with sequences from case 1 was 91.5%. No source for HEV transmission was identified, and the patient had not traveled abroad. Clinical outcome spontaneously improved within 2 weeks. HEV RNA test results 2 months postadmission were negative.

## Conclusions

In these 2 cases, the temporal association between acute hepatitis and neurologic signs and symptoms, atypical neurologic features, and exclusion of a large panel of other potential etiologies are incentive to consider HEV as a possible cause of meningitis, diffuse neuralgic pain, and polyradiculoneuropathy. The association of neurologic signs and symptoms with acute hepatitis E was previously reported for several patients (Table A1). Eight case-patients had Guillain-Barré syndrome (GBS) (*3**,**4**,**6**–**11*), which has been associated with a variety of bacterial or viral infections, including hepatitis viruses other than HEV (*10**,**11**,**15*). Four other reports described distinct clinical presentations of acute meningoencephalitis with seizure (*1*), Bell palsy (*2*), acute transverse myelitis (*12*), and neuralgic amyotrophy (*13*).

In most cases, only a few potential etiologies for GBS were tested for. In addition, in all but 2 reported cases (*3**,**4*), HEV diagnosis was based only on detection of anti-HEV IgM in serum. Dalton et al. detected HEV genotype 3 RNA in serum from a patient with acute hepatitis E in association with GBS, whereas HEV RNA testing of CSF was negative (*3*). Kamar et al. described polyradiculoneuropathy with central nervous system involvement in a kidney transplant recipient who had chronic hepatitis E 33 months after acute hepatitis E (*4*). In this case, HEV RNA was sequenced concurrently in serum and CSF at hospitalization for neurologic involvement. By contrast, the 2 cases in our report occurred concomitantly with acute hepatitis E. In the patient reported by Kamar et al. and in case-patient 1 in this report, HEV RNA load was ≈2 log lower in CSF than in serum (*4*). Rupture of the blood–brain barrier could have enabled passive entry of the virus into CSF. The negativity of HEV RNA testing in the CSF in case-patient 2 might be explained by lower titers in CSF than in serum because HEV RNA titer in serum was low. In the case reported by Kamar et al., phylogenetic analysis suggested that neurologic symptoms could be associated with emergence of neurotropic variants because clonal HEV RNA in CSF clustered apart from those found in serum. In case-patient 1 reported here, HEV sequences recovered from serum and CSF were 100% identical; nevertheless, they were compared on a short open reading frame 2 fragment (106 nt) and on samples collected at time of acute hepatitis E, whereas comparison was performed 33 months postacute hepatitis in the case reported by Kamar et al. Regarding the outcome of neurologic symptoms associated with acute hepatitis E, patients fully recovered within 1 and 24 weeks, except in the cases reported by Chalupa et al. (*10*) and Kamar et al. (*4*). In this latter case-patient, no improvement was noted after 4 months, and the patient died of decompensated cirrhosis.

Previous and current observations suggest that HEV diagnostic testing should be performed for patients who have concurrent neurologic symptoms and liver cytolysis. Further investigations are needed to assess the actual prevalence of HEV infections in cases of neurologic disorders, especially those of unknown etiology.

## Addendum

Kamar et al. recently reported neurologic complications in 7 (5.5%) patients among 126 with locally acquired acute and chronic HEV genotype 3 infection diagnosed during 2004–2009 at 2 hospitals in the United Kingdom and France (Kamar N, Bendall RP, Peron JM, Cintas P, Prudhomme L, Mansuy JM, et al. Hepatitis E virus and neurologic disorders. Emerg Infect Dis. 2011;17:173–9).

## Appendix

**Table A1:** Clinical features and laboratory findings for neurologic disorders associated with hepatitis E infection*

Reference and year	Country		Age, y/sex		Neurologic symptoms	ALT, IU/L	CSF		Other infectious etiologies excluded by serologic or molecular testing	Serum				CSF			Treatment/outcome
							Leukocytes, cells/mm^3^	Protein, g/L		Anti-HEV IgM	Anti-HEV IgG	HEV RNA		Anti-HEV IgM	Anti-HEV IgG	HEV RNA	
Cases from this study																	
Case 1, 2010	France		54/F		Meningitis with neuralgic pain: headaches, photophobia, diffuse paresthesia, and pain of upper and lower limbs	566	74	1.03	Serum: HAV, HBV, HCV, HIV, measles virus, rubella virus, HSV, VZV, EBV, parvovirus B19, dengue virus, *Leptospira* spp., *Brucella* spp., *Coxiella burnetii*, *Rickettsia conorii*, *R. typhi*, *R. felis*	+	+	+ Genotype 3		+	NT	+ Genotype 3	Total recovery in 2 wk
									CSF: CMV, HSV, VZV, EBV, HHV6, Toscana virus, mumps virus, measles virus, *Listeria monocytogene*s, *Mycobacteria* spp., *Mycoplasma* spp., all bacteria (16S ribosomal RNA detection)								
Case 2, 2010	France		49/M		Polyradiculoneuropathy: Proximal muscle weakness, paresthesia, and pain of upper and lower limbs	78	10	1.02	Serum: HAV, HBV, HCV, HIV, EBV, CMV, VZV, parvovirus B19, mumps virus, brucellosis, *Bartonella henselae, Bartonella quintana*	+	+	+ Genotype 3		–	NT	–	Clinical recovery in 2 wk
									CSF: EBV, HSV, VZV, enterovirus, Toscana virus, WNV, *Borrelia burgdorferi*, all bacteria (16S ribosomal RNA detection)								
Cases reported in the literature																	
Guillain-Barré syndrome:																	
(*6*), 2000		India		50/M	Generalized paresthesia, weakness of the lower limbs, hypotonia, areflexia	114	<5	1.86	Serum: HAV, HBV, HCV	+	NT	NT		NT	NT	NT	Improvement 2 wk after neurologic onset; total recovery within 1 mo
(*7*), 2002		India		35/M	Weakness of upper and lower limbs and of respiratory muscles	752	NT	NT	Serum: HAV, HBV, HCV, HDV, HIV	+	NT	NT		NT	NT	NT	IVIG; total recovery within 2 wk
(*8*), 2005		India		58/F	Muscular weakness, diffuse areflexia, left infranuclear facial palsy	1,448	<2	0.80	Serum: HAV, HBV, HCV	+	NT	NT		NT	NT	NT	IVIG, plasmapheresis; total neurologic recovery in 2 wk; tiver biochemistry normalized after about 5 wk
(*3*), 2008		United Kingdom		42/M	Paresthesia and weakness in lower limbs	632	NA	NA	NA	+	NA	+ Genotype 3		NT	NT	–	Total recovery after 3 mo
(*9*), 2009		Belgium		66/M	Progressive ataxia; weakness and paresthesia of legs, with ataxia and neuropathic pain	1,813	<5	1.72	Serum: HAV, HBV, HCV, HIV, VZV, CMV, EBV, HSV, adenovirus, *Campylobacter* spp.	+	NT	NT		NT	NT	NT	IVIG; progressive improvement of walking perimeter and pain within 4 mo
(*10*), 2010		Czech Republic		65/M	Loss of strength in upper limbs and partially in lower limbs; pain in both shoulders	1,920	7	0.7	Serum: HSV, EBV, CMV, HAV, HBV, HCV, enterovirus, inflluenza A/B virus, parainfluenza viruses 1–3, adenovirus, tick-borne encephalitis virus, *Borrelia burgdorferi*, *Anaplasma phagocytophilum,* *Chlamydia pneumoniae*, *Mycoplasma* spp.	+	+†	NT		NT	NT	NT	Persistence of residual tetraparesis 19 mo after onset (chronic demyelinating polyneuropathy); improvement after IVIG
									CSF: VZV,HSV, enterovirus, *A. phagocytophilum,* *C. pneumoniae*, *Mycoplasma* spp. *B. burgdorferi*.								
(*11*), 2010		Ireland		40/M	Progressive weakness of the legs; paresthesia of hands and feet, then quadriplegia, repiratory insufficiency, and facial weakness	57	<5	0.38	Serum: HAV, HBV, HCV, HIV, CMV, EBV	+‡	NA	NT		NT	NT	NT	IVIG, followed by plasmapheresis; total recovery within 6 mo
(*4*), 2010		France		44/M	Peripheral nerve involvement; proximal muscular weakness of the joints of the 4 limbs; bilateral pyramidal syndrome	105	7	0.76	Serum: HBV, HCV, HIV, CMV, EBV	+	+	+ Genotype 3		–	–	+ Genotype 3	IVIG; no improvement; death 1 mo later from decompensated cirrhosis
									CSF: CMV, HSV, VZV, JC virus, *Cryptococcus* antigen, *Toxoplasma gondii, Candida* spp								
Other neurologic diagnosis																	
(*1*), 2001		India		28/F	Meningoencephalitis: nuchal rigidity; drowsiness; desorientation; bilateral Babinski reflex and seizure	1,890	12	1.10	Serum: HAV, HBV, HCV, HIV, EBV, *Treponema pallidum*	+	+ at mo 6	NT		NT	NT	NT	Improvement of neurologic symptoms in 3 wk
(*12*), 2006		India		12/F	Acute transverse myelitis: weakness of lower limbs with sphincter dysfunction, pyramidal syndrome	NA	NA	NA	Serum: HAV, HBV, HCV, measles virus, rubella virus, HSV	+	NA	NT		NT	NT	NT	Total recovery within 10 d without treatment
(*2*), 2006		India		32/M	Bell palsy: right-sided lower motor neuron facial palsy	1,000	NA	NA	Serum: HAV, HBV, HIV, *T. pallidum*	+	NT	NT		NT	NT	NT	Total recovery in 3 wk
(*13*), 2009		United Kingdom		44/M	Bilateral neuralgic amyotrophy of upper limbs	2,547	NT	NT	Serum: HAV, HBV, HCV, *Borrelia burgdorferi, Treponema pallidum*, HTLV	+	NT	NT		NT	NT	NT	Resolution of pain 6 wk after onset; total recovery of sensory motor deficiency in 2 y

*ALT, alanine aminotransferase level; CSF, cerebrospinal fluid; HEV, hepatitis E virus; Ig, immunoglobulin; HAV, hepatitis A virus; HBV, hepatitis B virus; HCV, hepatitis C virus; HSV, herpes simplex virus; VZV, varicella zoster virus; EBV, Epstein-Barr virus; +, positive; NT, not tested; CMV, cytomegalovirus; HHV6, human herpes virus 6; –, negative; HDV, hepatitis D virus, WNV, West Nile virus; HDV, hepatitis D virus; IVIG, intravenous immunoglobulin; NA, not available; HTLV, human T-lymphotropic virus. †Two weeks after first anti-HEV IgM detection. ‡Weakly positive.
